# Some Remarks on the Treatment of Inflamed Pulp

**Published:** 1894-06

**Authors:** Frank C. Porter


THE
AMERICAN JOURNAL
—OF—
DENTAL SCIENCE.

VOL. XXVIII. BALTIMORE, JUNE, 1894. NO. 2.

ARTICLE I.

### SOME REMARKS ON THE TREATMENT OF INFLAMED PULP.

BY FRANK C. PORTER, M. R. C. S., L. R. C. P., L. D. S., D. D. S., ETC.

In choosing the treatment of inflammation of the pulp as the subject of my remark to-night, I do so because, personally, it is a subject of great interest to me, and, secondly, because it is one which is daily thrust upon our notice, being in many instances the exciting cause which sends the patient to his dentist.

Before proceeding to discuss the actual subject of my paper, I should like to remind you of the following points connected with the anatomy of the pulp, which have an all-important bearing on the pathology of its inflammation, and consequently of the treatment thereof.

1. There are, so far as is known, no lymphatics in the dental pulp. Obviously, then, should exudation from the vessels occur as the result of inflammation, the means whereby such a state of things would be remedied in other parts of the body is wanting. While on the subject of drainage it may be well to bear in mind the especial difficulty of getting rid of fluid of the pulp chambers of lower teeth on account of gravity tending to keep the fluid in the apical portion of the tooth.

2. The unyielding nature of the walls of the pulp cavity, causing its contents to feel the slightest increase of pressure severely.

3. The inaccessibility of the pulp for the easy and thorough application of sedative dressings to the diseased part. This at first sight may seem contrary to general experience. To clear out the superficial decay from a carious tooth and place a soothing dressing over an exposure, or over the region of the pulp is easy and simple enough. But the exposure is often minute and, in addition, has emanating from it serum or pus. The injured part of the pulp, on the other hand, is probably of considerable extent and runs far beyond the limit of the surface exposed, extending beneath the dense hard cavity wall. How, then, is the thorough application to be made?

The modes of treatment usually followed is either:—

I. Employment of means to secure the salvation of as a living organ.

II. Destruction of the pulp with the idea of its subsequent removal.

It is not my intention to consider separately the treatment of acute and chronic pulpitis, for clinically the same principles of treatment are applied to both, nor is it my intention to discuss the details of method. I want rather to view the successes and failures of one method as compared with those of the other, and consider circumstances which may contra-indicate the employment of one or other method.

Cases of traumatic exposure, where the operator has by accident drilled into the pulp through hard and healthy dentine, I leave out the question altogether, for the pulp in these cases is not inflamed beyond what ensues from the wound thus given.

For my own part, though I may be mistaken, as my experience is more limited than that of most gentlemen present, I have made up my mind, over and over again, that capping an exposed or inflamed nerve is, except under exceptional circumstances, a proceeding to be avoided if possible. My conviction intensifies as my experience grows.

Having so strongly condemned what, theoretically, is most in accord with modern conservative surgery, I will try to make clear some of my reasons for so doing.

Firstly, I maintain that capping is usually an altogether unsuccessful operation, for the pulp, in the majority of instances, dies sooner or later, in spite of the most careful mersures taken to ensure its salvation. To take a common example: A patient comes complaining of toothache. The offending tooth is examined and a cavity found. This is full of remains from several meals in various stages of decomposition acting as strong irritants, at the same time it is a very hot bed of micro-organisms. The cavity is syringed out and the superficial decay removed. A minute exposure is found, or perhaps the operator, owing to the sensibility of the tooth, refrains from excavating the softened dentine in the neighborhood of the pulp, leaving the latter, at any rate, unexposed to vision. A sedative dressing is applied and the patient told to come again in two days. Owing to the removal of the irritants and to the absorption of some of the dressing the pain subsides. On the second visit the operator caps the pulp, puts in a filling of low thermal conductivity, or, if he be a bold man, an amalgam or gold over this. The tooth probably remains quiet, but meantime one of two things is happening to the pulp. Either (*a*) resolution of the inflamed part takes place and a scar is formed with, perhaps, some so-called secondary dentine; or (*b*) (and this in the great majority of cases) the pulp dies a lingering death, the surroundings tending to favor such a termination in the following manner. Serum, and later pus, continuing to exude have no escape.

Lymphatics to carry it away there are none. Artificial drainage has been done away with, for the only outlet has been blocked by a solid plug. Pent up, the serum collects, tending to extend the inflammation by acting as a foreign body and irritant, and secondly tends to strangulation and thrombosis of the vessels by the pressure it exerts on their walls, until finally, as many of us know, the pulp becomes a stinking corpse. The trouble, however, is not over, for one fine day some putrescent matter finds its way through the apical foramen into the pericementum, and then—well! I leave the rest to your imagination, for there are few who have not, at some period of their lives, experienced the agony of an acute alveolar abscess. Truly, the last state of that tooth was worse than the first.

That a tooth with a healthy pulp is in many ways a more durable and serviceable organ than one without a pulp, I grant. It is true in some cases eburnation occurs, arresting the progress of further decay. It is true that pulpless teeth are often slightly loose, and are subject to slight attacks of periostitis when the general state of the system is run down, and that, as age advances, they are shed before those with healthy pulps. But, of all others, a tooth whose pulp has died, an acute alveolar abscess supervening, is much more subject to the disadvantages above named than one whose pulp has been killed surgically.

Take another very common class of cases. Interstitial cavities with an inflamed and exposed pulp. That the tooth may not feel the inconvenience of sudden thermal changes an osteo has been inserted. Grant, for sake of argument, the pulp lives. Everything is comfortable, and the patient, as patients will, forgets to come and have the tooth examined in a few months time. The osteo at the cervical margin is dissolved away, decay goes on underneath, and the state of the tooth at the end of a year is somewhat worse than it was at the beginning. On the other hand, had the pulp been destroyed, a permanent filling could have been inserted without any difficulty in the beginning. If the insertion of a permanent filling depends on the death of a pulp, I say by all means let the pulp die.

There is, however, a class of cases, akin to that just mentioned, for which I look upon destruction of the pulp as contra-indicated. I allude to those cases where pain has been felt in a tooth when sudden thermal changes have occurred in the mouth, or upon irritants coming in contact with it, but where, upon examination of the cavity, none of the usual signs of an exposure are discovered and when the dentine over the pulp is not very disorganized. In such cases nothing more than mere hyperæmia has occurred in the pulp chamber, and the chances are no septic matter has penetrated within its walls. Every facility, therefore, is present for the pulp to return to its normal state, upon means being taken to remove the irritation. It frequently happens that the dentist is often put into a quandary by a patient presenting himself with an exposed and inflamed pulp, stating that it is his intention to start on a holiday the following day. What is to be done? If it can be so arranged that the patient can pay a visit on the following day, I should unhesitatingly apply an arsenical dressing. The pulp, though probably not sufficiently devitalized to permit of its total extirpation, will have probably become painless enough to permit of partial extraction. An astringent antiseptic dressing can then be applied under a temporary filling. Even should the arsenic have not acted sufficiently to permit any of the pulp being removed, pain is less likely to recur during the weeks following its application, than if it had not been used.

Should, however, a second visit be impossible, the position and nature of the cavity would, to my mind, be the chief factors in deciding the mode of treatment to follow. If the cavity were such that a filling could be inserted over the dressing, feeling confident that no arsenic would ooze through and get to the gum, I should have no scruples about leaving the dressing in for some weeks, until such time as the operation could be completed. I have known the wonted small amount of arsenic to be left in teeth for weeks without any untoward result accruing. The amount used must be small and the filling securely inserted.

Certain circumstances render it expedient, however, that we should do all in our power to save the life of a pulp. Chief among these, may be mentioned, children who come under our treatment with an exposed and aching pulp in a tooth, which, though it be erupted, may not have its roots fully formed and calcified. Here, if the life of the pulp can only be prolonged for a few weeks, much progress may be made towards the tooth's formation. On the other hand, the case requires careful watching, for with enlarged apical foramina, increased facility for septic absorption upon death of the pulp is presented, with a consequent abscess of intensified severity. But, happily, the largeness of this foramen has a compensating advantage:—thrombosis of the supplying artery is less likely to happen, and death of pulp, therefore, less likely to follow.

There is one other point I should like to mention before closing, and that is the apparent inefficiency of arsenic with certain patients. This, I think, is due to the drug not being put into contact with tissue able to absorb it, and one of the following conditions is generally present:

(1) Inflammatory granulation tissue has formed on the exposed surface. This tissue as is well known absorbs drugs very sparingly.

(2) Death of the half of the pulp nearest the crown, the other half still being alive. Frequently I have noticed that when a pulp is in this state, an instrument coming in contact with the dead portion causes pain in the remainder of the organ. Thus it is an easy matter for an operator to be deceived in cases where a good view is not obtainable, and to wrongly imagine he is placing his dressing on living tissue.

If after two applications, it is impossible to get sufficient of the pulp away to warrant the chamber and roots being filled, I generally insert an osteo and wait for two or three months, when there is usually no difficulty in finishing the tooth.

There are, I feel, many things connected with this interesting subject that I have left untouched, and those I have touched upon I feel have been dealt with very superficially and imperfectly. If, however, I have interested you and provided food for discussion, I feel I shall not altogether have written in vain.—*London Dental Record.*

